# Seasonal Analysis of Match External Load in Hungarian Second-Division Professional Football Across Three Competitive Seasons Using GPS-Derived Match-Average Data

**DOI:** 10.3390/sports14040155

**Published:** 2026-04-15

**Authors:** Richárd Bauer, Bálint István Ruppert, Bálint Kilvinger, Árpád Petrov, István Barthalos, László Suszter, Ferenc Ihász, Zoltán Alföldi

**Affiliations:** 1 Doctoral School of Regional and Economic Sciences, Széchenyi István University, H-9026 Győr, Hungary; ruppert.balint99@gmail.com (B.I.R.); kilvingerbalint@gmail.com (B.K.); petrov.arpad@gmail.com (Á.P.); 2 Department of Health Promotion and Exercise Science, Széchenyi István University, H-9026 Győr, Hungary; barthalos.istvan@sze.hu (I.B.); ihasz.ferenc@ga.sze.hu (F.I.); alfoldi.zoltan@sze.hu (Z.A.); 3 Sport and Health Sciences Research Group, Eszterházy Károly Catholic University, H-3300 Eger, Hungary; suszter.laszlo@uni-eszterhazy.hu

**Keywords:** Catapult Vector S7, second division, acceleration, deceleration, Player Load^TM^

## Abstract

Background/Objectives: The aim of this study was to describe seasonal trends in match-average External Load (EL) variables across three (2022/23, 2023/24, 2024/25) consecutive competitive seasons in a Hungarian professional second-division soccer team (Gyirmót FC Győr), using the Catapult Vector S7 Global Navigation Satellite System (GNSS). Specifically, Average Distance (AD; m), Average Player Load^TM^ (PL; AU), and Acceleration–Deceleration Efforts (>2 m·s^−2^) (ADE) were examined. The study aimed to provide descriptive reference values and characterize seasonal variation in match EL demands within a professional second-division context. Methods: A descriptive seasonal comparison was conducted based exclusively on aggregated match average EL values. The unit of analysis was the match, with each match contributing one aggregated value per variable derived from players who completed the full match. A total of 94 matches were included (2022/23: N = 38; 2023/24: N = 29; 2024/25: N = 27); matches with red cards were excluded. EL data were collected using a 10 Hz Catapult Vector S7 GNSS. Results: The median AD decreased continuously from the 2022/23 season (10.210 m) to the 2024/25 season (9.795 m). The median PL decreased from 1002 (2022/23 and 2023/24) to 846 in the 2024/25 season. The median ADE decreased from 220.8 (2022/23) to 199.0 (2024/25). Conclusions: Lower values were observed across match EL variables, with the most pronounced reduction in PL. These findings provide descriptive reference values and may support the interpretation of seasonal variation in match EL demands in professional second-division soccer.

## 1. Introduction

In soccer, External Load (EL) refers to a player’s physical movements, which are expressed through various running speed zones, accelerations, and decelerations [1]. With the widespread use of Global Positioning System (GPS) technology, these demands can be monitored during both matches and training sessions [2]. Modern tracking systems are based on Global Navigation Satellite System (GNSS) technology, which includes multiple satellite constellations (e.g., GPS) and enables accurate tracking of players’ positions on the field, typically at higher sampling frequencies (e.g., 10 Hz). GNSS represents a broader positioning framework integrating multiple satellite constellations; therefore, GNSS-based systems may provide improved signal quality and positional accuracy compared with GPS-only systems [3]. Monitoring EL has become essential in modern soccer to objectively quantify match demands and support evidence-based load management strategies [4]. GPS technologies also play a key role in the EL analysis of various team sports by measuring the physical demands on players, such as in field hockey [5], handball [6], basketball [7], and rugby [8,9]. In soccer, 10 Hz GNSS devices have demonstrated improved accuracy in measuring instantaneous speed and movement patterns compared with lower-frequency systems, supporting their suitability for EL monitoring in dynamic match environments [10,11]. The Catapult Vector S7 system, combining high-frequency GNSS and inertial sensor technology, has been shown to provide acceptable validity and reliability in measuring locomotor and mechanical EL variables in team sports [12].

Total distance covered is one of the most frequently used EL metrics for assessing the workload of players during training and competition, measured in absolute terms (in meters) [13,14,15,16]. Elite athletes typically cover 10–14 km during a full match [17,18,19]. However, total distance should be interpreted within the context of playing position and match conditions, as central midfielders typically cover greater distances, while forwards perform more high-speed and transitional actions, and overall EL is influenced by tactical and situational factors [20]. Match EL values (e.g., total distance) are strongly influenced by playing position, competition level, and tactical context, with reported distances varying substantially at 9–12 km for centre-forwards, and evidence shows that lower divisions may present either lower demands (e.g., Brazilian leagues) or even higher total distances (e.g., in the English Championship and League One) compared to English Premier League, emphasizing the importance of contextual interpretation [21]. In addition to distance-based metrics, Player Load^TM^ (PL), derived from tri-axial accelerometer data, is frequently used to quantify cumulative mechanical load experienced during match play [22,23,24,25,26]. PL reflects the sum of accelerative movements across multiple planes and is expressed in arbitrary units (AU) [27]. PL is widely used in soccer to track players’ EL, and higher PL values have been associated with greater fatigue, muscle damage, and increased injury risk [28,29]. Previous research indicates that PL is also position-dependent, with central midfielders typically exhibiting higher values than players at other positions due to their greater involvement in multidirectional activity [30]. However, PL is based on a proprietary algorithm, and its lack of transparency, sensitivity to signal noise, and limited standardization across devices should be considered when interpreting results [31]. Acceleration and deceleration actions represent another key component of match EL, given the frequent, high-intensity speed changes required during competition [32]. Previous research has highlighted that many non-contact injuries occur during acceleration and deceleration phases, emphasizing the importance of monitoring these high-intensity efforts [33]. Acceleration and deceleration demands are also position-dependent, with wide players and forwards typically performing more high-intensity actions, whereas central midfielders accumulate higher overall volumes, and central defenders generally present lower values [34]. In both research and applied settings, acceleration-based metrics are typically defined using absolute thresholds, most commonly >2 m·s^−2^ or >3 m·s^−2^, to classify high-intensity actions [34,35,36,37]. However, it should be considered that the accuracy of 10 Hz GPSs may decrease at very high acceleration magnitudes (e.g., >4 m·s^−2^), which may influence the interpretation of acceleration-derived variables [38].

In professional sports, the demand for objective, sport-specific data is evident in the abundance of GPSs available on the market today; however, their widespread adoption is often limited to elite levels due to the prohibitive cost of high-quality devices, leaving the integration of GPS technology in amateur and semi-professional team sports in its infancy owing to constrained financial resources [39]. Although numerous studies have described seasonal variations in EL in elite soccer, most research has focused on a single season or short-term observation periods [39,40,41,42,43]. Consequently, it remains unclear how match EL evolves across consecutive seasons when assessed consistently within the same team, particularly in lower-tier professional environments. Furthermore, descriptive analysis of match EL provides valuable insight into the physical demands imposed on players completing full-match duration, supporting practitioners in identifying load profiles and designing targeted training strategies to adequately prepare players for competition.

Therefore, this study focused exclusively on the match-average value across consecutive seasons. Accordingly, the aim of the present study was to describe and interpret seasonal trends in match EL variables using the Catapult Vector S7 GNSS across three consecutive competitive seasons for a Hungarian professional second-division soccer team. Specifically, we examined Average Distance (AD; m), Player Load^TM^ (PL; AU), and Acceleration–Deceleration Efforts (>2 m·s^−2^) (ADE). By applying a consistent multi-season methodological approach within the same team context, this study seeks to characterize seasonal patterns in aggregated match EL and provide practically relevant insights to support applied decision-making in load monitoring and training management.

## 2. Materials and Methods

### 2.1. Participants

We designed the study as a season-level descriptive comparison based on EL aggregated data. All data were collected from the same Hungarian professional second-division team (Gyirmót FC Győr) across three consecutive competitive seasons. A total of 94 matches were included in the analysis (2022/23: N = 38; 2023/24: N = 29; 2024/25: N = 27). We included only matches in which players completed the full playing time and excluded matches involving red cards to ensure consistent playing conditions.

For each match, we calculated a single aggregated match-average value for each EL variable. The unit of analysis was a match, and all analyses were performed exclusively at the match level using aggregated values. Individual player-level data were not analyzed separately. Accordingly, our findings reflect season-level differences in aggregated match EL values rather than within-player longitudinal changes. We acknowledge that the lack of positional distribution data limits the interpretation of seasonal differences. EL variables are position-dependent in soccer; however, the analysis was conducted at the team level using aggregated match-average values, without positional stratification. Therefore, position-specific analyses were not performed. This limitation is acknowledged. A total of 33 male professional soccer players contributed data during the study period. We report descriptive anthropometric characteristics (mean ± SD) as follows: age, 24.68 ± 4.17 years; height, 183.21 ± 7.19 cm; body mass, 78.99 ± 8.12 kg. These characteristics are presented for descriptive purposes only and were not included in the statistical analyses. The study was conducted in accordance with the guidelines and regulations of the Scientific and Research Ethics Committee of Széchenyi István University (protocol code SZE/ETT-94/2022 (XII.5.), approval date 5 December 2022) and the Declaration of Helsinki.

### 2.2. Procedures and Data Collection

EL data was collected using the Catapult Vector S7 GNSS device (Catapult Sports, Melbourne, Australia), operating at a sampling rate of 10 Hz. In addition, data quality indicators also confirmed the accuracy of GNSS recordings. The horizontal dilution of precision (HDOP) was 0.83 ± 0.10, with an average of 14.92 ± 0.93 satellites connected during data collection. The overall average GNSS signal quality was 69.86% ± 3.09%, which is consistent with values reported in previous research [12,44]. Devices were worn in a custom vest positioned between the scapulae during official matches [45]. The EL variables analyzed were aggregated averages calculated for each match (N = 94): Average Distance (AD; m), Average Player Load^TM^ (PL; AU): A proprietary metric from Catapult representing the cumulative load from accelerations in three planes (anterior–posterior, medial–lateral, and vertical), calculated as the square root of the sum of squared instantaneous rates of change in acceleration divided by 100 [46]; and Acceleration–Deceleration Efforts (>2 m·s^−2^) (ADE), defined as the total count of acceleration events >+2 m·s^−2^ and deceleration events <−2 m·s^−2^ during the match. Acceleration and deceleration were quantified using the Catapult Sports Ltd., Melbourne, AustraliaCatapult OpenField software (version 4.7) and the associated Catapult Cloud platform. The >2 m·s^−2^ threshold corresponds to the manufacturers’ default settings and was not modified by the authors. Data was downloaded and processed using the manufacturer’s software (OpenField, Console software (version 3.15.2; Catapult Sports Ltd., Melbourne, Australia) Catapult Sports) immediately post-match to ensure data integrity.

We acknowledge that the use of a fixed absolute threshold (>2 m·s^−2^) to define acceleration and deceleration efforts was not specifically validated for professional second-division soccer. Although similar thresholds have been reported in methodological and applied monitoring contexts, consistent evidence in professional match settings remains limited [34,35,36,37,38]. Therefore, the use of fixed thresholds should be interpreted with caution, as they may not reflect individual physical capacities or contextual match demands. In addition, this study focused on selected EL variables and did not include other commonly used metrics, such as high-speed running or sprint distances. While these variables were chosen to reflect general movement and mechanical demands, they represent only part of the total EL of a match. Accordingly, the results should be interpreted within the context of these specific variables rather than as a comprehensive assessment of overall match EL.

### 2.3. Statistical Analysis

Match-average EL metrics were summarized across seasons using mean, standard deviation, median, and interquartile range, and are presented in tabular form. The distribution of EL metrics across seasons was visually examined using boxplots, including median values, interquartile ranges, and individual data points. These visualizations were used to illustrate seasonal trends and variability in match EL metrics. All results are presented descriptively, and no inferential statistical analyses were performed. Descriptive statistical analysis was performed using JASP (Version 0.19.3 JASP Team, Amsterdam, The Netherlands).

## 3. Results

**Figure 1:**
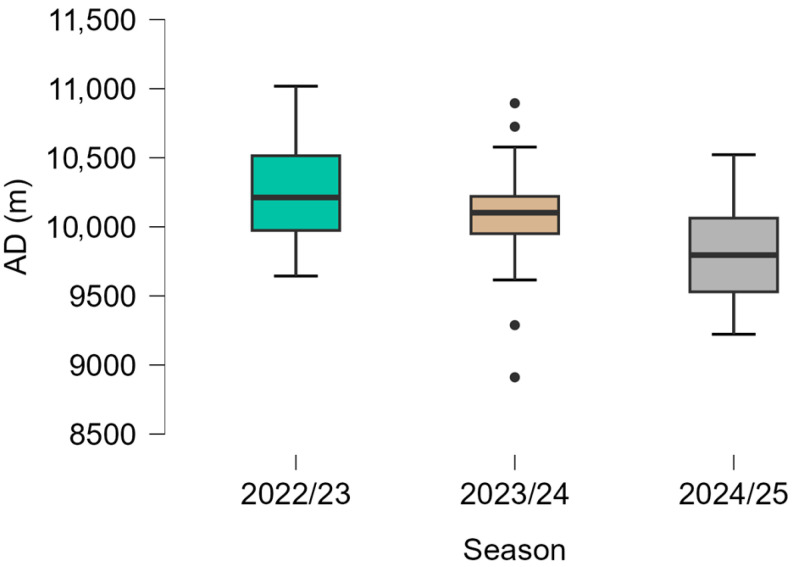
Distribution of Average Distance (AD) across three consecutive seasons (2022/23, 2023/24, 2024/25). Boxplots indicate the median and interquartile range, with outliers shown as individual points.

**Figure 2:**
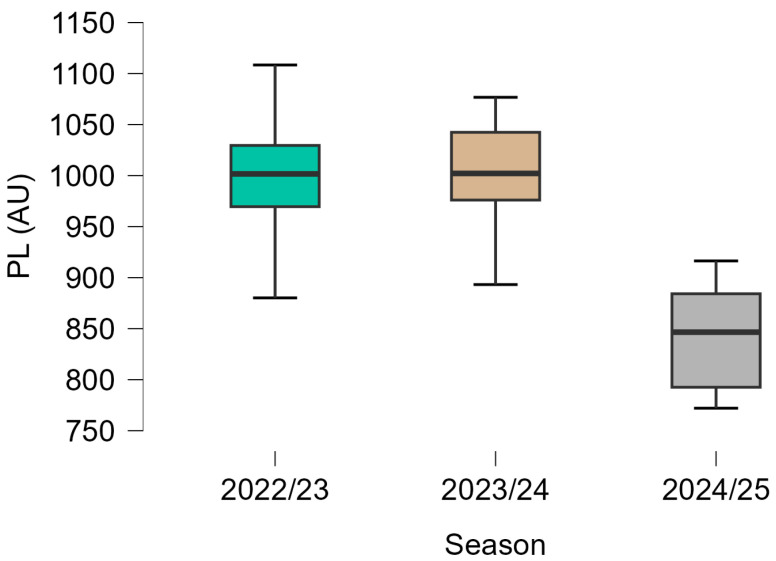
Distribution of Player Load^TM^ (PL) across three consecutive seasons (2022/23, 2023/24, 2024/25). Boxplots indicate the median and interquartile range.

**Figure 3:**
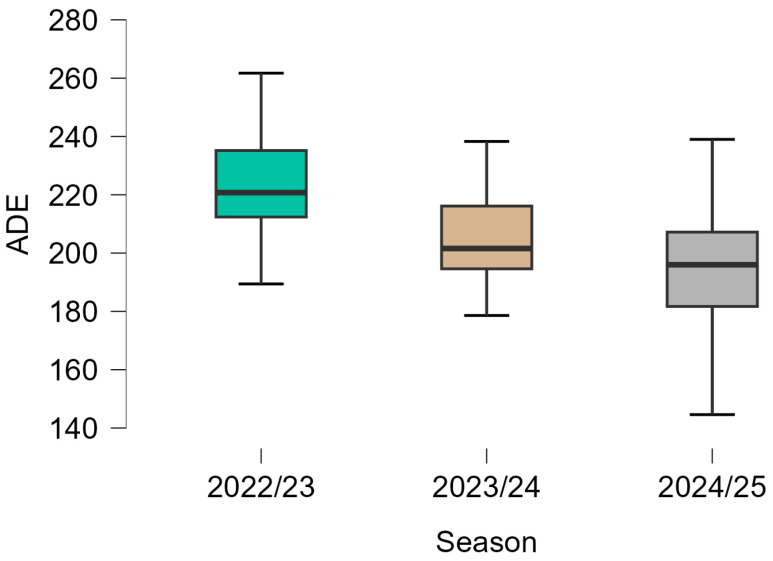
Distribution of Acceleration–Deceleration Efforts (>2 m·s^−2^) (ADE) across three consecutive seasons (2022/23, 2023/24, 2024/25). Boxplots indicate the median and interquartile range.

Descriptive statistics for EL variables across the three seasons are presented in Table 1, and their distributions are illustrated in Figure 1, Figure 2 and Figure 3.

Lower median AD values were observed across the three seasons. The highest values were recorded in the 2022/23 season, followed by lower values in 2023/24 and 2024/25. The distribution of values, as shown in Figure 1, indicates moderate variability within each season, with a slightly narrower interquartile range in the final season. A similar pattern was observed for PL, with higher values in the 2022/23 and 2023/24 seasons and lower values in 2024/25. Figure 2 shows that the distribution of PL values was relatively consistent in the first two seasons, whereas lower median values and reduced overall range were observed in the final season. ADE values were also lower in the later seasons compared with 2022/23. The highest median values were observed in 2022/23, followed by lower values in 2023/24 and 2024/25. As illustrated in Figure 3, variability remained moderate across seasons, although occasional lower values were observed, particularly in the later seasons.

## 4. Discussion

The present study describes seasonal variation in match EL across consecutive competitive seasons for a Hungarian professional second-division soccer team using aggregated EL data. Lower values were observed across seasons in AD, PL, and ADE. Given the descriptive design and aggregated EL dataset, these findings should be interpreted as showing seasonal variation in match EL. Multi-season descriptive match EL analyses remain limited in the literature. Most available studies have examined single-season or short-term in-season variation in EL, primarily at the elite level, which limits long-term comparisons across competitive cycles [39,40,47]. Previous research has demonstrated that match running distance may fluctuate across competitive periods due to contextual and tactical factors, including match congestion, pacing strategies, and training periodization [48,49,50]. In addition, the physical EL experienced during a match is influenced by contextual and tactical factors, including player positions, tactical formations, the state of the match, and the league’s characteristics. Different match demands have been reported at various competitive levels, reflecting the interaction between roles and contextual match conditions. In second-division soccer, the physical exertion during a match may also be influenced by the greater variability of tactical formations, match tempo, and competition structure compared to top-tier leagues [21].

AD decreased progressively from the 2022/23 season through 2024/2025 (Figure 1). This presents an overall reduction of approximately 400 m in median match distance across the three seasons (Table 1). The match AD values observed in the present study (~9.8–10.2 km) are consistent with previously reported ranges in professional and semi-professional soccer [17,19]. Match running distances in adult male soccer typically range from 10 to 12 km, depending on competitive level, tactical context, and match characteristics [51]. The seasonal reduction observed in AD remained within this commonly reported range, indicating that the observed values fall within established ranges of locomotor match demands. However, because AD reflects overall locomotor volume rather than multidirectional mechanical load characteristics, it should be interpreted alongside complementary mechanical indicators when examining seasonal EL variation [30,31,32,33,34,35,36,37,38,39,40,41,42,43,44,45,46,47,48,49,50,51,52].

PL showed the largest seasonal difference among the examined variables, decreasing from ~1000 AU to 840 AU across the three seasons (Figure 1). PL remained relatively stable in 2022/23, followed by a marked decrease in 2024/25. The reduction between the first and third seasons was approximately 155–160 AU in central tendency values. According to a previous study, match PL values typically range between ~800 to 1000 AU, depending on playing position, tactical involvement, and competitive level, with higher values observed in central positions due to greater multidirectional and transitional movement demands [30]. As an accelerometer-derived metric integrating movement across multiple planes, PL reflects cumulative mechanical load rather than locomotor volume alone [22,31]. Previous studies have highlighted that PL is sensitive to changes in movement intensity, direction changes, and mechanical demands during match play [27]. The magnitude of PL reduction observed in the present study, therefore, provides internally consistent descriptive reference values within the context of the present dataset. Given the lack of established contextual thresholds for meaningful seasonal change in PL, these differences should be interpreted cautiously and not assumed to reflect changes in physiological status.

ADE also showed a gradual decrease across seasons, though with greater variability than AD and PL. Median values decreased from 220.8 efforts in 2022/23 to 201.6 in 2023/24 and 196.0 in 2024/25, corresponding to an overall reduction of approximately 25 efforts across the study period (Table 1). In the present study, we used a fixed absolute threshold (>2 m·s^−2^) to define acceleration and deceleration events, providing a consistent reference value across seasons. Comparison of ADE values across studies remains challenging due to methodological variability in defining and quantifying acceleration and deceleration events. Previous reviews emphasize that there is currently no universal consensus regarding threshold selection, filtering methods, or typical match counts for high-intensity acceleration and deceleration actions in soccer [35,43]. This lack of standardization limits direct comparison between datasets.

Seasonal comparisons were conducted across competitions with different numbers of matches due to structural differences in the competition format. Although all seasons were fully competitive, varying sample sizes may affect the stability of seasonal estimates. All EL variables examined showed gradual reductions across the three seasons, most evident in the 2024/25 season, with the largest shift observed in PL, followed by ADE and AD. These findings indicate seasonal variation in match EL and are consistent with previously reported patterns in team-sport literature; however, no causal inferences can be made regarding the underlying mechanisms [49]. While descriptive differences were observed across seasons, the practical implications of these changes should be interpreted cautiously, as no established contextual benchmarks for meaningful change in these EL variables are currently available. Given the descriptive and aggregated nature of the dataset, these findings represent seasonal variation in match EL within a professional second-division team context. Multi-season EL datasets collected using consistent methodology within the same team remain scarce in the literature, particularly outside elite top-tier leagues. In this context, the present findings may provide useful reference values for longitudinal monitoring.

From an applied perspective, the results provide descriptive reference values for match AD, PL, and ADE within a professional second-division context. These values may help practitioners contextualize seasonal variation in match EL when implementing longitudinal monitoring strategies. From a research perspective, the study highlighted the need for further multi-season investigations incorporating positional data, contextual match variables, and internal load measures to better understand the factors influencing seasonal EL variation. Greater methodological consistency in defining acceleration and deceleration thresholds would also improve comparability across studies. Overall, the present findings contribute to the limited body of literature reporting multi-season match EL data in professional soccer and provide internally consistent reference values for longitudinal comparison within similar competitive environments.

Beyond the contextual and tactical factors previously discussed, seasonal variation in match EL may also be associated with differences in how training load (TL) is distributed relative to match demands across competitive periods. The structure of weekly TL and exposure to high-intensity actions has been suggested to be associated with variations in EL characteristics [48,53]. Furthermore, integrative monitoring frameworks emphasize that EL variables should be interpreted within a broader system that incorporates positional roles, contextual match characteristics, and tactical organization [34]. From this perspective, seasonal shifts in AD, PL, and ADE represent changes in measured match EL across seasons; however, their underlying causes may be related to broader adjustments in training distribution, competitive structure, or tactical organization, which were not examined in the present study and should therefore be considered as potential hypotheses.

Because the present multi-season analysis relied exclusively on aggregated descriptive statistics and did not include training–match load relationships, positional stratification, contextual indicators, or internal load measures, the contributors to the observed reductions could not be determined. Accordingly, the seasonal decreases should be interpreted strictly as descriptive longitudinal patterns within one professional team context.

## 5. Conclusions

This study describes seasonal variation in match EL across three consecutive competitive seasons in a Hungarian professional second-division soccer team using aggregated EL data. Lower values were observed in AD, PL, and ADE across the examined seasons, with the most pronounced decline in PL, reflecting seasonal differences in cumulative mechanical match demands. While the present study does not establish a causal mechanism, these descriptive findings may provide reference values for season-to-season monitoring in applied settings. These results highlight how season-to-season monitoring of match EL can reveal variation across seasons in locomotor and mechanical load indicators. Given the descriptive design and the absence of contextual or internal load measures, the observed differences should be interpreted cautiously as showing seasonal patterns within a single team. Nevertheless, the present results contribute to the limited literature reporting multi-season match EL data in professional soccer and provide reference values for monitoring in similar competitive environments.

## 6. Limitation

The study has several limitations. First, the sample size was relatively small, as the analysis included 94 matches. Although this dataset is representative of the Hungarian second-division context, it does not reflect higher divisions or the international level. An additional methodological limitation concerns the unit of analysis. All analyses were conducted at the match level using aggregated match-average values (N = 94), rather than individual player-level longitudinal data. Although this approach reduces within-match dependency, several players contributed data across multiple seasons. Therefore, complete independence of observations cannot be assumed, and the findings should be interpreted as descriptive seasonal comparisons rather than within-player longitudinal changes. Furthermore, several potentially influential contextual variables were not controlled for, including weather conditions, pitch surface, opponent strength, technical and tactical data, and individual player characteristics (e.g., age, playing position, injury history). These factors may influence EL variables during matches. The number of matches also varied across seasons due to structural changes in the league format: 20 teams (38 matches) in 2022/23, 18 teams (34 matches) in 2023/24, and 16 teams (30 matches) in 2024/25. Additionally, matches involving red cards were excluded to ensure comparable playing conditions. Consequently, the smaller sample size in the 2024/25 season reflects structural differences rather than incomplete data collection. However, the reduced number of matches may influence the precision of seasonal estimates, limit direct comparability across seasons, and potentially bias the observed seasonal trends. Given the dataset’s aggregated and descriptive nature, the results should be interpreted as showing team-level seasonal patterns rather than precise estimates of player-level changes. We did not use inferential statistical modeling; therefore, no conclusions can be drawn regarding statistical significance or causal relationships. Future research using larger datasets and player-level longitudinal data may benefit from applying mixed-effects or other hierarchical modeling approaches to better capture within-player seasonal dynamics.

## Figures and Tables

**Table 1:** Descriptive statistics of EL variables across the three competitive seasons (2022/23–2024/25). Values are based on EL aggregated averages (N = 94 matches).

	AD (m)			PL (AU)			ADE		
	2022/23	2023/24	2024/25	2022/23	2023/24	2024/25	2022/23	2023/24	2024/25
N	38	29	27	38	29	27	38	29	27
Median	10,210.00	10,100.00	9795.00	1002.00	1002.00	846.60	220.80	201.60	196.00
Mean	10,220.00	10,070.00	9833.00	1000.00	1002.00	842.20	223.10	206.50	195.20
Std. Deviation	353.30	400.70	378.10	46.10	48.01	47.25	16.83	16.04	21.66
IQR	540.50	269.90	533.50	60.02	66.40	91.61	22.81	21.53	25.50

Abbreviations: AD = Average Distance (m); PL = Player Load^TM^; ADE = Acceleration–Deceleration Efforts (>2 m·s^−2^); AU = Arbitrary Units; IQR = interquartile range (Q3–Q1), representing the middle 50% of the data. Values are presented as EL aggregated averages.

## Data Availability

The original contributions presented in the study are included in the article, further inquiries can be directed to the corresponding author.

## Abbreviations

The following abbreviations are used in this manuscript:

AD	Average Distance (m)
ADE	Acceleration–Deceleration Efforts (>2 m·s^−2^)
AU	Arbitrary Units
EL	External Load
GNSS	Global Navigation Satellite System
GPS	Global Positioning System
PL	Player Load^TM^
TL	Training Load
